# Seismic collapse assessment of bridge piers constructed with steel fibers reinforced concrete

**DOI:** 10.1371/journal.pone.0200072

**Published:** 2018-07-10

**Authors:** Yutao Pang, Lingxu Li

**Affiliations:** Faculty of Engineering, China University of Geosciences, Wuhan, China; Technische Universiteit Delft, NETHERLANDS

## Abstract

Steel fiber is one of the most widely used reinforcements to improve the performance of concrete members. However, few studies have been proposed to study the seismic performance of bridge piers constructed with steel fiber reinforced concrete. This paper presents the collapse vulnerability assessment of typical single bridge piers constructed with steel fibers. Fiber element models of RC bridge piers with and without steel fibers are firstly built by selecting suitable cyclic constitutive laws of steel fiber reinforced concrete, and then calibrated using the experimental results. The seismic capacity and inelastic demand of RC piers with steel fibers are quantified using both nonlinear static pushover analyses and nonlinear incremental dynamic analyses (IDA). In order to conduct the IDA, a suite of 20 earthquake ground motions are selected and scaled to different levels of peak ground acceleration (PGA). Collapse fragility curves are then generated using the maximum drift ratio of piers as the engineering demand parameter (EDP). In order to investigate the impact of various parameters on the collapse fragility curves, six parameters are considered in the parametric study: peak compressive strength of concrete, yield strength of steel, longitudinal reinforcement ratio, axial load ratio, transverse hoops ratio and steel fiber content. It was observed that the concrete strength, longitudinal reinforcement ratio and steel fiber content could significantly affect the collapse fragility curve of the bridge piers with steel fibers.

## Introduction

Bridges play a key role in a highway transportation network since they are the necessary and important link elements of the economic development circles in a country. A large number of reinforced concrete (RC) highway bridges in China were constructed due to the rapid development of the economy in the past 30 years. Meanwhile, many strong earthquakes have occurred in the last decades not only in China, but also throughout the globe. Recent major earthquakes (i.e. 1994 Northridge (USA), 1995 Kobe (Japan) and 2008 Wenchuan (China) earthquakes) caused severe damages to RC highway bridges. The main reason is due to the insufficient seismic capacity of bridge piers. The poor reinforcement details and low confinement of concrete in the bridge piers may lead to low ductility capacity and low shear strength, which can easily cause the brittle failure [1], [2] or flexural failure [3] and even the collapse of bridge piers. If a highway bridge damages severely or collapses in the strong earthquakes, the only option is to construct a new one, which is often expensive and time consuming. Comparing with many post-earthquake strengthening methods (such as the concrete or steel plate jacketing), it seems more economy and convenient to design a bridge pier with adequate seismic capacity to resist the seismic loading. Thus, the aim of the present paper is to achieve adequate deformation capacity of piers at the seismic design stage of highway bridges.

In last decades, the use of multi-phase composite materials has emerged as a viable approach for improving the performance of concrete elements. Among them, steel fiber reinforced concrete (SFRC) is a very promising material in which short steel fibers are uniformly distributed in the concrete to enhance the performance of conventional reinforced concrete. For the last two decades, SFRC has been successfully applied in a variety of applications, such as pavements and overlays, airport runways and structures [4], [5], due to the cost-effective features. For example, when the fraction volume of steel fiber is 1%, the steel fibers in the concrete will be 3.2 kg/m^3^, which will increase the costs ¥ 20.8 per m^3^ for SFRC bridge piers. If construction methods for SFRC are assumed to be mature which do not cause additional costs, the increasing percentage of the cost will be 6.1%, which is economy for practical engineering. Numerous researchers have found that the SFRC cannot only improve the compressive, tensile, shear and flexural strength of concrete member [6], [7], [8], [9], [10], but also enhance the ductility capacity, toughness and energy absorption ability in dynamic loading [11], [12], [13], [14].

There are some works dealing with the feasibility of fiber-reinforced concrete for improving the seismic performance of structures. Filiatrault et al. [15] conducted the quasi-static tests of three full-scale interior beam-column joints of a prototype building. The experimental results indicated that steel fibers could increase the shear strength of joints and diminish the requirements for closely spaced ties in the code. Lee et al. [16] investigated the benefits of the application of steel fibers to the brittle behavior of RC columns under seismic excitations. From the test results, it is found that the optimum steel fiber volume fraction is 1.5% for the maximum enhancement of shear strength. Zhang et al. [17] evaluated the effect of steel fibers on the seismic capacity of hollow rectangular piers. The study showed that the ductility capacity of hollow bridge piers is improved by steel fibers, and that the effect of steel fibers is similar to effect of the transverse hoops for seismic design. Zhang et al. [18] conducted the seismic fragility analyses of eight bridge piers, made of SFRC and conventional RC. The fragility results indicated that the seismic vulnerability of the bridge piers decreases with the increase of steel fiber content in a certain range. However, no works deal with the damage states and seismic collapse assessment of SFRC bridge piers, which are crucial for the future seismic risk evaluations of the bridge systems with SFRC piers.

In this paper, seismic performance of a new bridge pier constructed with steel fibers at design stage is investigated through both nonlinear static pushover analyses (NSPA) and incremental dynamic analyses (IDA). Fiber element models of bridge piers with and without steel fibers are established and validated with results of quasi-static cyclic tests. The flexural damage states are obtained by the NSPAs. Seismic collapse assessment of SFRC bridge piers are conducted through IDA analysis. The present paper focuses on quantifying the inelastic demand and capacities of SFRC bridge piers with hollow sections. The results of this study will be useful for the bridge designers for developing new strategies to improve the seismic capacity of current bridge systems.

## Configuration of bridge pier

The bridge piers in our study are built based on the experimental study [17]. In the experimental study, the bridge piers adopted are regarded as full-scale specimens. Thus, same dimensions of bridge piers are used in this work. The configuration of different SFRC bridge piers with hollow sections are illustrated briefly in this section. The SFRC bridge piers with hollow sections are assumed to be located in Sichuan Province, China. All the piers have identical dimensions and reinforcing details. The dimension of prototype pier is 500×360×1240 mm with a 100 mm section thickness. The dimension of the foundation block is 1200×1200×480 mm and loading block 600×600×360 mm. In order to identify the parameters which affect the seismic behavior of the SFRC bridge piers most, six parameters are considered in the parametric study: the compressive strength of concrete, *f*_c_, the yield strength of steel, *f*_y_, longitudinal reinforcement ratio, *q*_l_, axial load ratio, P, transverse hoop ratio, *q*_t_, and steel fiber content, V_f_. Table 1 lists the details of variable parameters considered in this paper. Table 2 illustrates the material properties of steel fibers reinforced concrete. It should be noted that the bridge piers adopted in this work is related to the typical piers in the practice engineering of bridges according to the following three aspects: a) the longitudinal reinforcing details including the yield strength of steels and longitudinal reinforcing ratio, b) the transverse reinforcing details containing the yield strength of stirrups, transverse hoops ratio and the space of stirrups; c) axial load ratio. The axial load ratio for typical highway bridge pier is among 10%-20%. Thus, in this paper, two levels are selected for SFRC bridge piers, 10% for the normal level and 20% for the high compression level.

**Table 1:** Details of variable parameters in this paper.

Variable parameters	Value		Units
	Level 1	Level 2	
Compressive strength of concrete, *f*_c_	21	34	Mpa
Yield strength of steel, *f*_y_	225	335	Mpa
Longitudinal reinforcement ratio, *q*_l_	1.5	2.5	%
Axial load ratio, P	10	20	%
Transverse hoop ratio, *q*_t_	0.5	1.5	%
Steel fiber content, V_f_	1	2	%

**Table 2:** Material properties of steel fibers reinforced concrete.

Material	Diameter (mm)	Length (mm)	Density (g/cm3)	Tensile/compressive strength (Mpa)	Elastic modulus (Gpa)
Concrete	-	-	3.3	34	30
Longitudinal steel	10	-	7.8	335	200
Hoop steel	6	-	7.8	235	200
Steel fibers	0.55	35	7.8	1143	200

**Fig 1:**
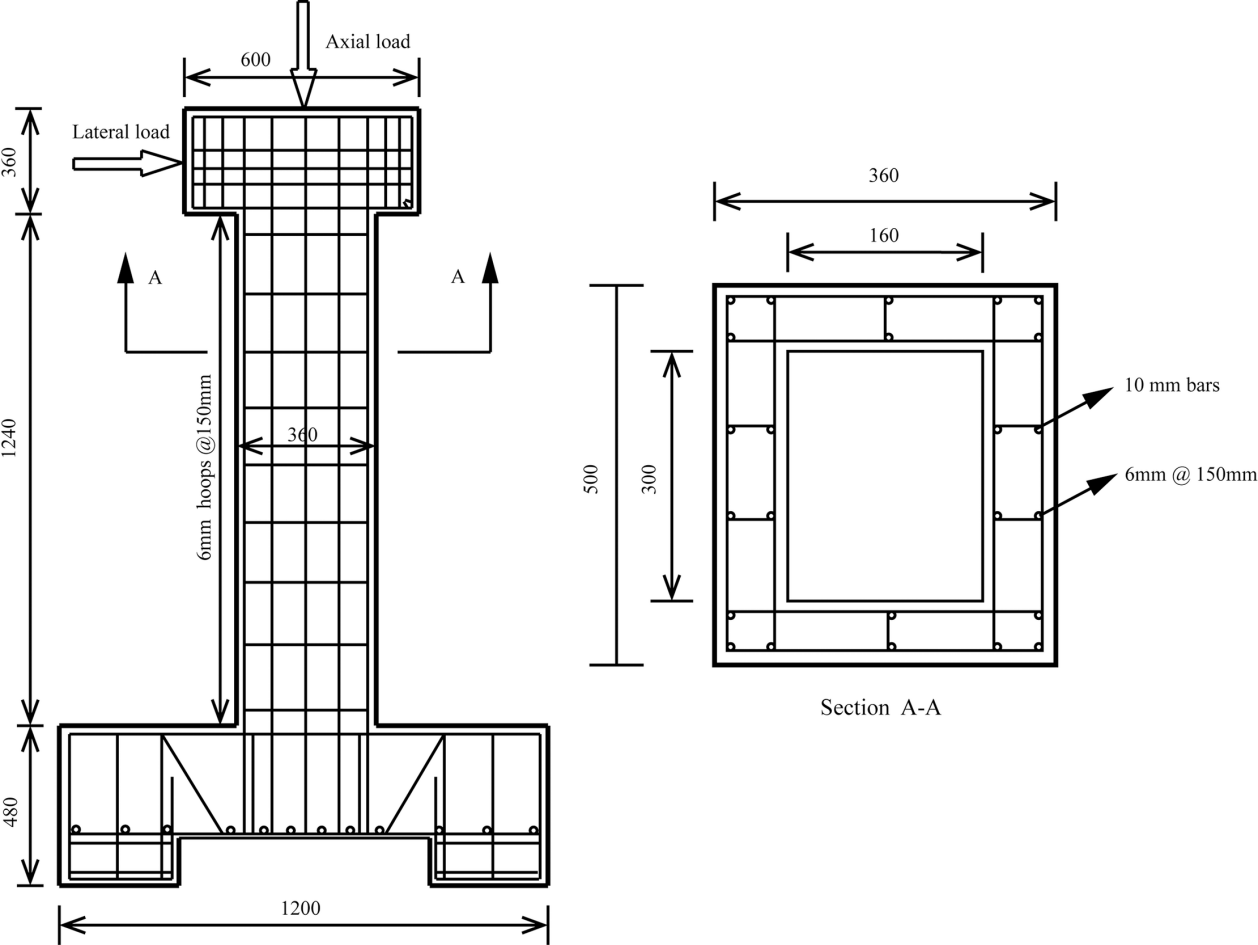
Configuration and reinforcement detailing considered in this study.

Table 3 illustrates the summary of the SFRC piers considered in this paper. Fig 1 shows the configuration and reinforcement detailing of the SFRC pier. 12 SFRC bridge piers with hollow sections are designed to investigate the effect of different parameters on the flexural damage limit states and seismic collapse fragility curves of SFRC bridge piers. Steel bars with 10 mm diameter are used as longitudinal reinforcements. Rectangle stirrups with diameter 6 mm are designed with 150 mm spacing as the transverse reinforcement in all piers. In Table 3, it should be noted that for each pier ID, only one parameter is changed and the others are kept constant, which means that the interaction effect of parameters are neglected.

**Table 3:** Summary of the SFRC bridge piers.

Variables	Pier-ID	*f*_c_(Mpa)	*f*_y_ (Mpa)	*q*_l_(%)	P(%)	*q*_t_(%)	V_f_.(%)
Strength of concrete, *f*_c_	P-1-34	34	335	2.5	0.2	1.5	2
	P-1-21	21	335	2.5	0.2	1.5	2
Yield strength of steel, *f*_y_	P-2-335	34	335	1.5	0.2	1.5	2
	P-2-235	34	235	1.5	0.2	1.5	2
Longitudinal reinforcements ratio, *q*_l_	P-3-2.5	34	235	2.5	0.1	1.5	1
	P-3-1.5	34	235	1.5	0.1	1.5	1
Axial load ratio, P	P-4-20	21	335	2.5	0.2	0.5	2
	P-4-10	21	335	2.5	0.1	0.5	2
Transverse hoops ratio, *q*_t_	P-5-1.5	34	335	2.5	0.1	1.5	1
	P-5-0.5	34	335	2.5	0.1	0.5	1
Steel fiber content, V_f_.	P-6-2	21	235	2.5	0.2	1.5	2
	P-6-1	21	235	2.5	0.2	1.5	1

## Numerical simulation

### 3.1 Finite element model of piers

**Fig 2:**
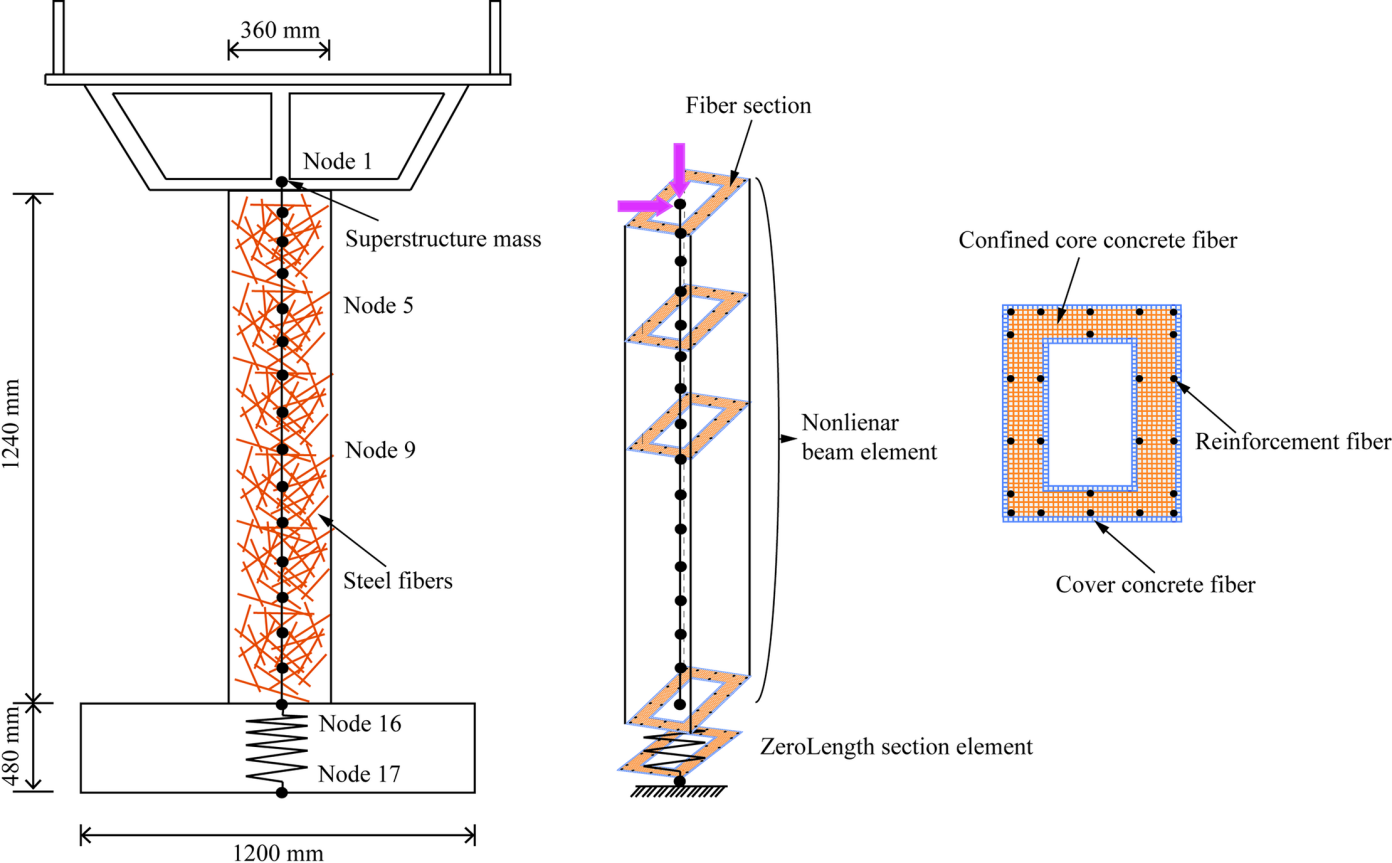
Schematic of fiber-based beam-column element model and section discretization.

**Fig 3:**
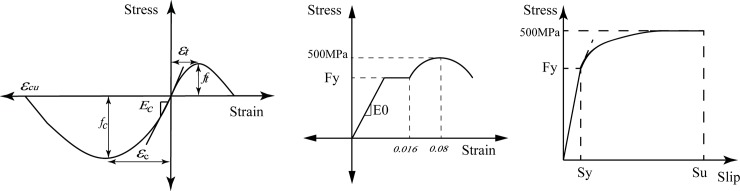
Stress-strain curves of the material model in the numerical models.

The 3D finite element model of SFRC bridge piers shown in Fig 1 and Table 3 is built using the open-source software OpenSees [19]. Fig 2 shows the schematic figure of fiber-based beam-column element model and section discretization. It should be noted that the mass of the superstructure is lumped at the top of the pier. In order to take the nonlinearity of materials into account, 3D fiber-based displacement-based beam-column elements with 5 numerical integration points have been used for modeling of the piers. To develop the hysteretic relationship of the pier sections correctly, each section are divided into 400–600 fibers to represent cover concrete, core concrete and longitudinal reinforcements. Each fibers have the suitable uniaxial state of stress: a) the Chang and Mander's concrete model (concrete07 model) with simplified unloading and reloading curves is used to model the cyclic behavior of concrete; b) the Chang and Mander reinforcing steel model is applied to model the longitudinal reinforcements, including the mechanical effects of strain softening, compression buckling and tensile fracture of the steel bars; c) the effect of fatigue is considered based on the Coffin-Manson equation for plastic strain amplitude. Fig 3 depicts the stress-strain curves of the material models in the numerical models. The strength of core concrete is calculated by considering the confinement effect of steel fibers using the following equations [20]:

$$f_{c}=f_{c0}+2.1604R_{I}$$ (1)

$$\varepsilon_{c}=\varepsilon_{c0}+0.0006R_{I}$$ (2)

$$R_{I}=W_{f}L_{f}/D_{f}$$ (3)

in which *f*_c0_ is the compressive strength of normal concrete; *ε*_c0_ is the corresponding concrete strain; *ε*_c_ is the strain of SFRC; R_I_ is the influence factor; W_f_ is the steel fiber mass content; *L*_f_ and *D*_f_ are the length and diameter of steel fiber respectively. In this study, the adopted steel fibers are RC-65/35-BN DRAMIX steel fibers, which have a diameter of 0.55 mm and length of 35 mm.

In order to account for the effect of steel fibers which increases the peak bond strength and reduces the bond degradation under seismic loading [17], a nonlinear rotational link element (zero-length element) with bond-slip rotations (bond_slip01 model) is employed at the pier-foundation interface. The modification of two properties *S*_y_ and *S*_*u*_ = 35*S*_*y*_ in this bond_slip01 model is used to represent the effect of steel fibers. The parameter, *S*_y_, can be calculated using the equation proposed by Harajli et al. [21] as following:

$$S_{y}=S_{1}e^{1.8[{\left(u_{y}/u_{m}(SFRC)\right)}^{2}-1]}$$ (4)

$$u_{y}=0.78\sqrt{f_{c}}{\left(\frac{c+0.45cV_{f}L_{f}/D_{f}}{d_{b}}\right)}^{2/3}$$ (5)

$$u_{m}(SFRC)=(1+0.34\sqrt{V_{f}L_{f}/D_{f}-0.25})u_{m}(RC)$$ (6)

$$u_{m}(RC)=0.75\sqrt{f_{c}}{\left(c/d_{b}\right)}^{2/3}$$ (7)

in which *S*_y_ is the reinforcing bar slip at member interface under yield stress; u_y_ is yielding bond stress; c is minimum concrete cover; S_1_ = 0.15*c*_0_, *c*_0_ is the clear distance between the lugs of the reinforcing bar; d_b_ is reinforcing bar diameter; u_m_ is maximum bond stress; V_*f*_ is steel fiber content.

### 3.2 Model calibration

The numerical finite element model is calibrated using the experimental studies by Zhang et al. [17]. The dimension of prototype pier is 500×360×1240 mm. The longitudinal steel has a diameter of 10 mm and the transverse steel has a diameter of 6 mm. The stirrups have 150 mm spacing. The clear cover of the concrete is 30 mm. The pier is free on top and has an axial load of 184.8 kN representing for 10% of the axial capacity. The lateral loading is applied at the loading block in the cyclic load test. The compressive strength of the unconfined concrete, the yield strength of longitudinal and transverse steel bars are 34, 335 and 310Mpa.

**Fig 4:**
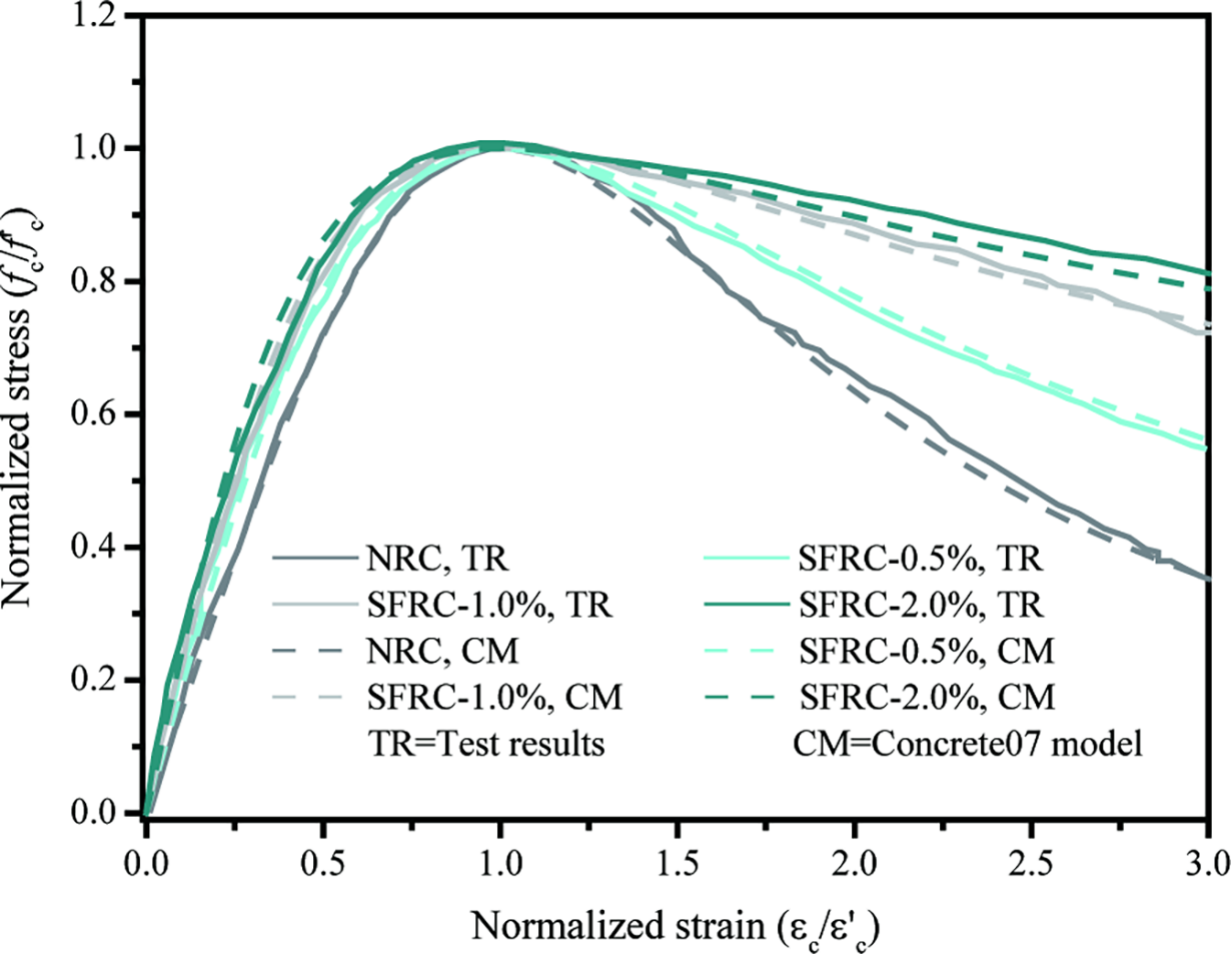
Comparison between the concrete07 model and test results [22].

**Fig 5:**
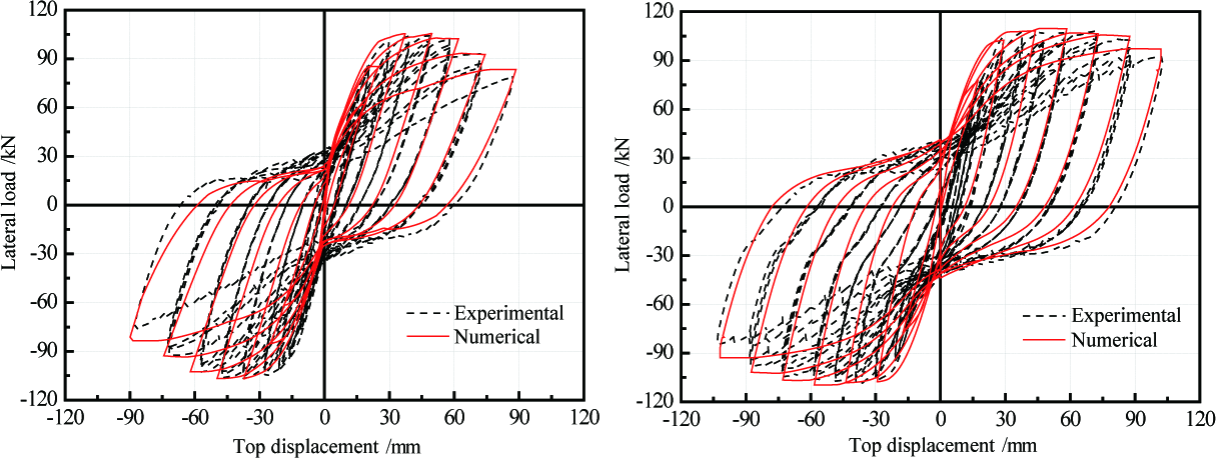
Comparison of the FE models and experimental results of RC pier and pier with steel fibers.

The test data of stress-strain curves in the study [22] are used to calibrate the concrete07 model adopted in this work. Fig 4 shows the comparison between the Concrete07 model and test results [22]. From the Fig 4, it can see that the used material model in the software OpenSees can simulate the behavior of SFRC. In order to calibrate the finite element model, the piers with steel fibers is built and modeled using the open-source software OpenSees under the displacement controlled method. The cyclic loading history is same as the experimental test [17]. In the analysis, the displacement controlled method has been used for both normal RC and SFRC piers with an increment of 1 mm until the maximum displacement 100 mm reaches. Fig 5 shows that the relationship of lateral load and top displacement of normal RC pier and pier with steel fibers respectively. As shown in Fig 5, the finite element model in the present paper can provide the initial stiffness, post stiffness and ultimate stress of normal RC and SFRC piers with a good accuracy when comparing to the experimental results.

### Nonlinear static pushover analysis

**Fig 6:**
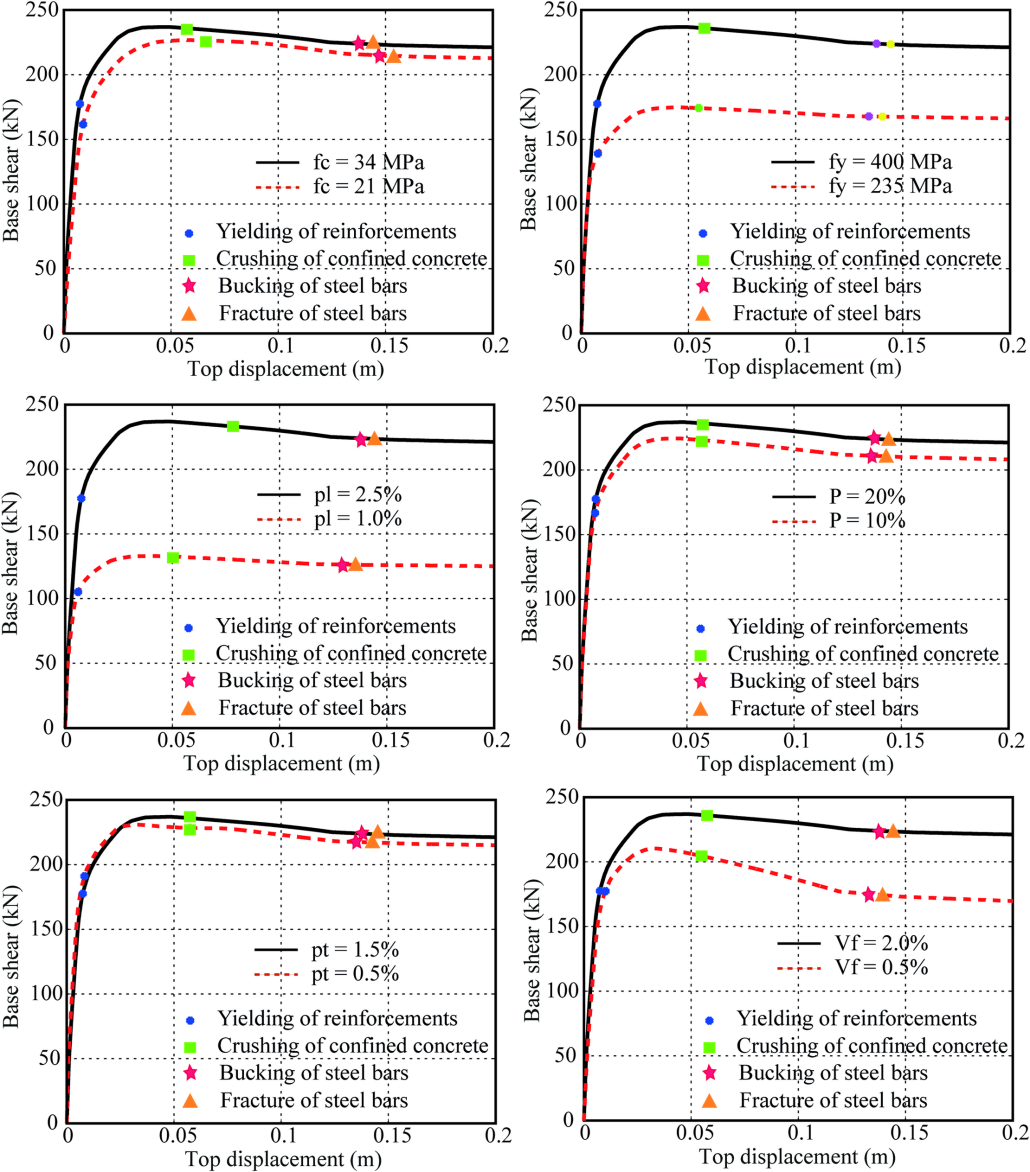
Results of nonlinear static analyses considering the effect of different parameters.

The seismic performance of bridge piers can be evaluated in terms of the flexural limit states. The flexural limit states can be reflected by the strain limits of longitudinal reinforcements. Four strain limits of longitudinal reinforcements are considered as four performance criteria: a) first yielding of longitudinal steels, which can be calculated as the ratio of yielding force and elastic modulus; b) first crushing of core concrete (while the crushing strain of core concrete changes normally from 0.015 to 0.05 [3], the present paper uses 0.045 as the reference value); c) first buckling of longitudinal reinforcements and d) first fracture of longitudinal reinforcements. The buckling and fracture of longitudinal steel bars can be predicted and determined as functions of the effective confinement ratio by the Berry and Eberhard equations [23]. In the current study, the tensile strain of longitudinal steel bars in the hollow section reaches 0.055 and 0.057, to represent for the buckling and fracture of longitudinal steel bars. In order to investigate the effect of different modeling parameters on the flexural limit states of SFRC bridge piers, various nonlinear static pushover analyses are conducted. Fig 6 shows the effect of different modeling parameters on the flexural limit states of SFRC bridge piers. Table 4 illustrates the base shear and displacement at four limit states. Table 5 depicts the relative difference of base shear and displacement at yielding, crushing, buckling and fracture limit states.

**Table 4:** Base shear and displacement at yielding, crushing, buckling and fracture limit states.

Variables		Pier-ID	Yielding of reinforcements		Crushing of confined concrete		Buckling of steel bars		Fracture of steel bars	
			Disp (m)	Base shear (kN)	Disp(m)	Base shear(kN)	Disp (m)	Base shear (kN)	Disp (m)	Base shear (kN)
Strength of concrete		P-1-34	0.0137	191.0	0.0623	236.1	0.1421	224.5	0.1486	224.0
		P-1-21	0.0121	166.5	0.0626	227.2	0.1508	215.9	0.1576	215.4
Yield strength of steel		P-2-335	0.0064	120.9	0.0572	167.2	0.1363	159.2	0.1426	159.0
		P-2-235	0.0063	105.9	0.0509	129.4	0.131	124.8	0.1364	124.7
Longitudinal steel ratio		P-3-2.5	0.0069	151.6	0.0619	222.6	0.1413	210.9	0.1479	210.5
		P-3-1.5	0.0051	103.1	0.0571	153.2	0.1358	144.8	0.1421	144.6
Axial load ratio		P-4-20	0.0065	165.8	0.0619	227.2	0.1465	216.3	0.1536	216.0
		P-4-10	0.0062	145.7	0.0613	215.9	0.1437	205.4	0.1505	205.0
Transverse hoops ratio		P-5-1.5	0.0078	155.5	0.0558	196.5	0.1331	165.1	0.1394	164.3
		P-5-0.5	0.0072	155.3	0.0552	190.8	0.129	163.3	0.1357	162.4
Steel fiber content		P-6-2	0.0107	151.8	0.0737	222.8	0.1597	211.2	0.1669	210.9
		P-6-1	0.0109	149.6	0.0675	197.1	0.1492	170.3	0.1559	169.5

**Table 5:** Relative difference of base shear and displacement at yielding, crushing, buckling and fracture limit states.

Variables	Yielding of reinforcements		Crushing of confined concrete		Buckling of steel bars		Fracture of steel bars	
	Disp (m)	Base shear (kN)	Disp(m)	Base shear(kN)	Disp (m)	Base shear (kN)	Disp (m)	Base shear (kN)
Strength of concrete	11.7%	12.8%	-0.5%	3.7%	-5.8%	3.8%	-5.7%	3.9%
Yield strength of steel	1.6%	12.4%	11.0%	22.6%	3.9%	21.6%	4.3%	21.5%
Longitudinal steel ratio	26.1%	32.0%	7.8%	31.2%	3.9%	31.4%	3.9%	31.3%
Axial load ratio	4.6%	12.1%	1.0%	4.9%	1.9%	5.0%	2.0%	5.1%
Transverse hoops ratio	7.7%	0.1%	1.1%	2.9%	3.1%	1.1%	2.7%	1.2%
Steel fiber content	-1.8%	1.4%	8.4%	11.5%	6.6%	19.4%	6.6%	19.6%

It can be seen in Fig 6 and Table 5 that different parameters have different influence on the seismic capacity of SFRC bridge piers. For compressive concrete strength, it can be observed that the concrete compressive strength 21 MPa decreases the base shear at the yield and crushing limit states by 12.8% and 3.7% respectively, while the base shear at the buckling and fracture limit states decreases by 3.8% and 3.9% comparing to 34 MPa concrete strength. Moreover, the concrete compressive strength 21 MPa increases the displacement by 11.7% at the yield limit states, but decreases the displacement by 0.5%, 5.8% and 5.7% at the crushing, buckling and fracture limit states. This can be due to the reason that the steel fibers are more effective in the concrete with low compressive strength. Comparing to the high compressive strength, the low compressive strength has lower modulus, which exhibit lower pier stiffness, and thus have much higher deformability as well as larger displacement. For yield strength of steels, the higher yield steel strength (335 MPa) has much higher base shear by 12.4%, 22.6%, 21.6% and 21.5%, and larger displacement by 1.6%, 11.1%, 3.9% and 4.3% at four limit states when comparing to the lower yield steel strength (235 MPa). The yield strength of steels will affect the flexural capacity of bridge piers, which may increase the base shear significantly. But the stiffness of pier does not change much by the yield strength of steels, which has a little influence on the top displacement. For longitudinal steel ratio, it can be seen that it can affect the base shear and displacement capacity of bridge piers significantly at four limit states. For base shear, the yielding, crushing, buckling and fracture capacity can be improved by 32.0%, 31.2%, 31.4% and 31.3% when the bridge pier uses 2.5% longitudinal reinforcement ratio. For displacement, the yielding, crushing, buckling and fracture capacity can be improved by 26.1%, 7.8%, 3.9% and 3.9%. It can be due to the reason that the increase of 1% longitudinal steel ratio can only increase the flexural capacity of bridge piers, but also exhibit larger pier stiffness. For axial load ratio, the base shear capacity at 10% axial load ratio decrease by 12.1%, 4.9%, 5.0% and 5.1%, while the displacements at different limit states are nearly the same for both 10% and 20% axial load ratios. This is due to the reason that the increase of axial load ratio has a larger influence on the flexural strength of bridge pier, while it does not change the stiffness of pier too much. For transverse hoops ratio, it can be observed that the maximum effect of transverse hoops ratio on the base shear capacity of SFRC piers is almost 3%. The yielding, crushing, buckling and fracture displacements are also improved by 7.7%, 1.1%, 3.1% and 2.7%, respectively. Although the transverse hoops ratio can affect the compressive strength of confined concrete, this effect is relatively small. Therefore, it can be concluded that the transverse hoops ratio has minor influence on the seismic performance of SFRC bridge piers. For steel fiber content, it can be observed that the steel fiber content significantly affects the flexural performance of the SFRC bridge piers. It shows 1.4%, 11.5%, 19.4% and 19.6% larger yielding, crushing, buckling and fracture base shear capacities comparing to the 1% steel fiber content. Similar trends can be found for the displacement at yielding, crushing, buckling and fracture limit states. This found is consistent with the previous studies that the steel fibers can not only improve the shear and flexural strength of structures, but also enhance the ductility (displacement) capacity.

## Incremental dynamic analysis

Comparing to the nonlinear static pushover analysis, nonlinear time-history analysis can provide most accurate estimation of seismic responses of bridge piers. In order to predict the structural responses in large nonlinear range, Luco and Cornell [24] and Vamvatsikos and Cornell [25] proposed the incremental dynamic analysis (IDA) method. IDA requires various nonlinear time-history analyses of a finite element model of a specific structure at different levels of intensities of ground motions. The aim of IDA is to estimate the accurate behavior of nonlinear seismic responses of the structure under seismic loadings. The selected levels of intensity measure (IM) of ground motions in the IDA needs to cover the entire range of structural responses, from elastic behavior through yielding to dynamic instability. The results of IDA are present in terms of IDA curves, which reflect the relationship between the engineering demand parameter (EDP) of the considered structure and IM of ground motions.

In this paper, IDA is carried out using the finite element models of piers with superstructure mass at the top. The superstructure mass is calculated by the properties of bridge deck, girder, parameter, and pavement, which is 18.48t in this study. PGA is chosen as the IM in the current paper in order to implement the scaling procedures conveniently. In order to cover the entire range (elastic, yield and inelastic) of seismic responses of bridge piers, PGA of the selected ground motions is scaled from 0.1 g to 4.0 g to give a large range of IM levels. The maximum drift of top pier can be monitored during the IDA and used as an EDP to generate the IDA curves. In general, when the maximum drift ratio of the top piers reaches a certain point, the finite element model will experience globe instability with a large and unreal displacement. In this paper, this point is considered as collapse point [26]. The collapse due to the numerical dynamic instability can provide a good understanding of the seismic behavior and performance of SFRC bridge piers. The corresponding PGA can be used for development of collapse fragility curves.

### 5.1 Selection of ground motions

Due to the limited number of available earthquake records in Sichuan Province, China, a suite of 20 as-record ground motions are selected from the PEER strong ground motions database is selected to account for the uncertainties and variability of ground motions. These ground motions are chosen based on the following rules: a) Vs30 index varies from 600 m/s to 700m/s to stand for the soil class between D and E; b) Moment magnitude ranges from 5 to 8 and hypocentral distance is between 10 km and 50 km to represent for the seismic hazard of local site; and c) PGA are in the range of 0.1–1.0g to avoid the earthquake ground motions with too small intensity. Table 6 gives the lists of earthquakes and corresponding information of the ground motions. It is assumed that the selected suite of ground motions can be applied as the representation of an earthquake motion in China. In this paper, one horizontal component of ground motion records is used as seismic input in the IDA analysis of bridge piers.

**Table 6:** Lists of ground motions used in IDA.

No.	Earthquake Name	Year	Earthquake Station	Magnitude	Hypocentral Distance	Vs30 (m/s)	PGA (g)	PGV (cm/sec)
1	'Imperial Valley-06'	1979	'Cerro Prieto'	6.53	26.74	659.6	0.154	18.39
2	'Hector Mine'	1999	'Hector'	7.13	30.38	684.9	0.336	37.02
3	'Duzce, Turkey'	1999	'Lamont 531'	7.14	31.07	659.6	0.159	12.66
4	'Chi-Chi, Taiwan'	1999	'TCU138'	7.62	25.5	652.9	0.196	40.66
5	'Chi-Chi, Taiwan-06'	1999	'TCU129'	6.3	36.81	664.4	0.341	16.50
6	'Coyote Lake'	1979	'Gilroy Array #6'	5.74	9.12	663.3	0.452	51.54
7	'Loma Prieta'	1989	'San Jose—Santa Teresa Hills'	6.93	26.66	671.8	0.273	25.69
8	'Chi-Chi, Taiwan'	1999	'TCU045'	7.62	77.91	704.6	0.604	44.12
9	'Kocaeli, Turkey'	1999	'Gebze'	7.51	49.68	792	0.238	51.97
10	'Chi-Chi, Taiwan'	1999	'WNT'	7.62	16.27	664.4	0.960	69.16
11	'Loma Prieta'	1989	'UCSC'	6.93	24.05	714	0.374	12.04
12	'Victoria, Mexico'	1980	'Cerro Prieto'	6.33	35.48	659.6	0.628	31.30
13	'Northridge-01'	1994	'Santa Susana Ground'	6.69	22.83	715.1	0.233	14.34
14	'Loma Prieta'	1989	'Gilroy—Gavilan Coll.'	6.93	33.84	729.7	0.294	30.79
15	'Chi-Chi, Taiwan-03'	1999	'TCU138'	6.2	29.79	652.9	0.133	19.72
16	'Loma Prieta'	1989	'UCSC Lick Observatory'	6.93	23.93	714	0.406	17.69
17	'Northridge-01'	1994	'LA Dam'	6.69	21.1	629	0.576	77.09
18	'Northridge-01'	1994	'LA 00'	6.69	22.67	706.2	0.382	22.07
19	'Northridge-01'	1994	'LA—Chalon Rd'	6.69	22.99	740.1	0.193	18.57
20	'Chi-Chi, Taiwan'	1999	'TCU129'	7.62	16.27	664.4	1.013	60.16

### 5.2 IDA analysis and IDA curves

**Fig 7:**
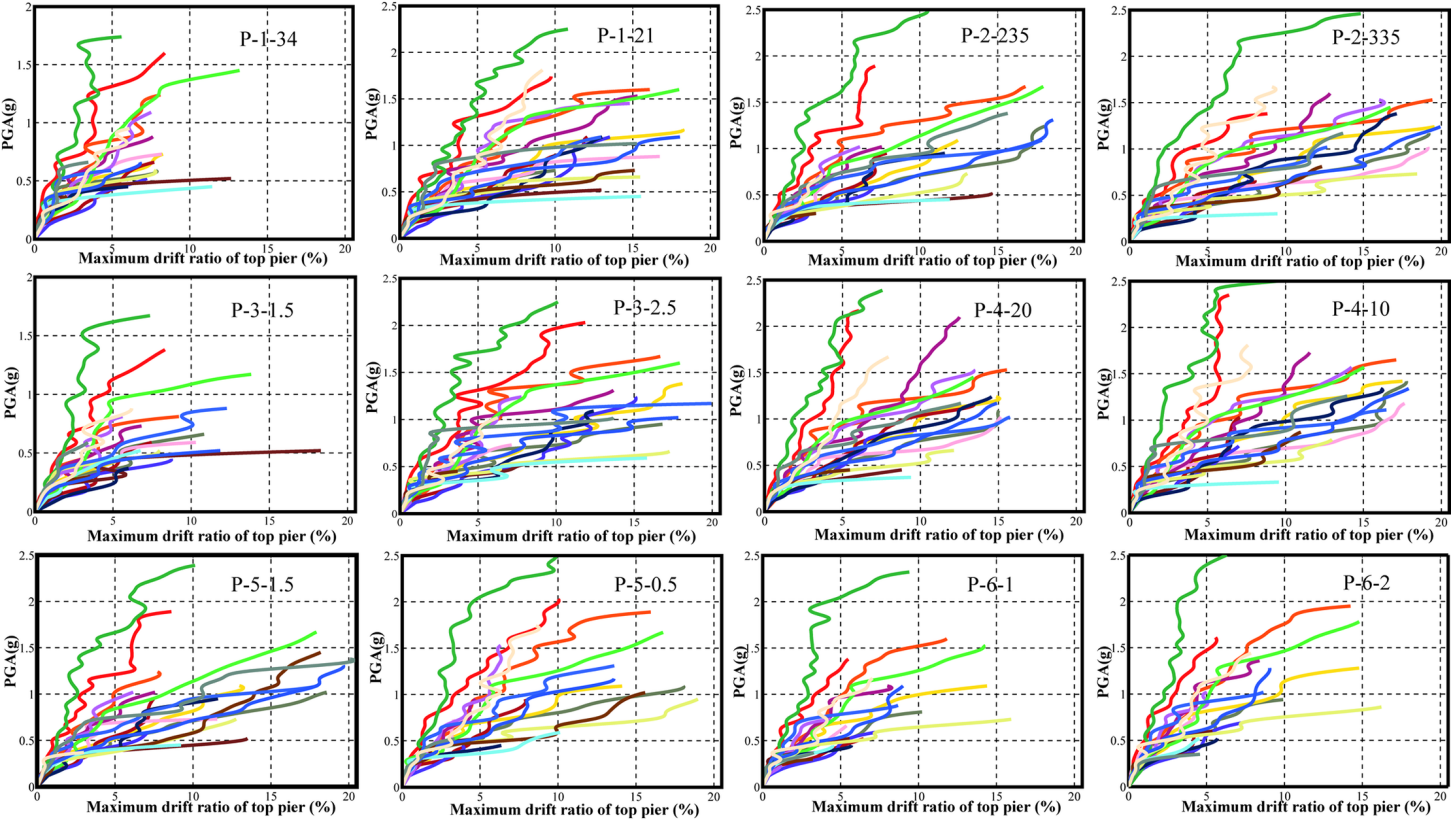
IDA curves of SFRC piers.

**Fig 8:**
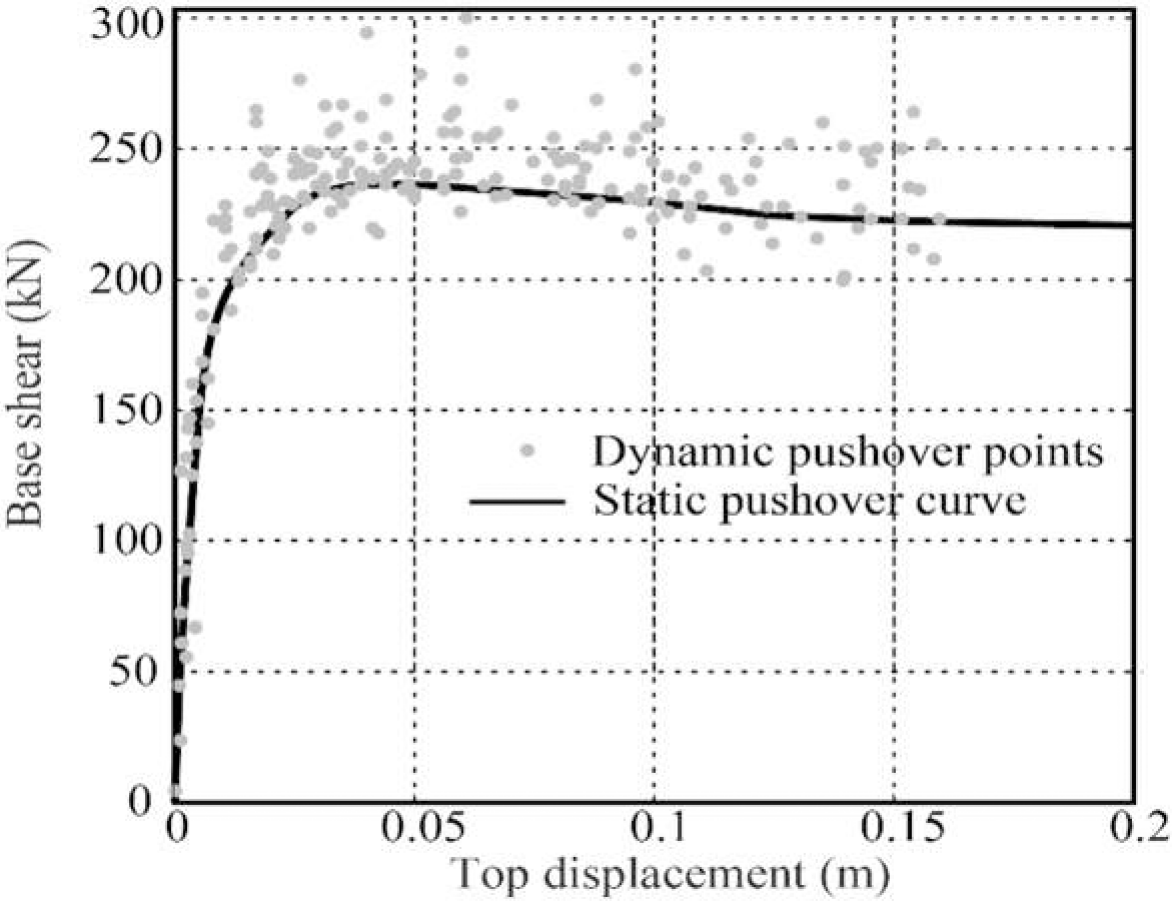
Comparison between the dynamic pushover points and static pushover curves.

For the SFRC bridge piers, inelastic seismic responses are estimated by conducting IDA analysis. IDA curves are then generated in terms of the relationships between IM and EDP as shown in Fig 7. Fig 7 depicts the IDA curves of all SFRC piers with different modeling parameters (Table 2). These IDA curves are generated using numerous nonlinear time-history analyses. In these analyses, 20 ground motion records are scaled to multiple levels of intensities. In this study, the ground motions are scaled based on the PGA which varies from 0.01g to 4g with an increment 0.01g. The IDA analysis is calibrated through the generation of dynamic pushover points from IDA curves, which are used to compare with the static pushover curves. Fig 8 shows this comparison using the bridge pier labeled as P-1-34 in the Table 4. It can see from Fig 8 that the dynamic pushover points coincide with the static pushover curves very well.

IDA curves combined with different modeling parameters are used to identify the collapse points which reflect the corresponding IM values. It can be seen from the Fig 7 that for different ground motions, the collapse points of numerical models can be different. For different parameters, the effect of each parameter on the IDA curves will be different. Thus, it is necessary to generate the collapse fragility curves of each bridge pier to see the effect of each parameter. The collapse points from IDA curves are summarized in S1 Table, which can be used to generate the collapse fragility curves.

## Collapse fragility curves

**Fig 9:**
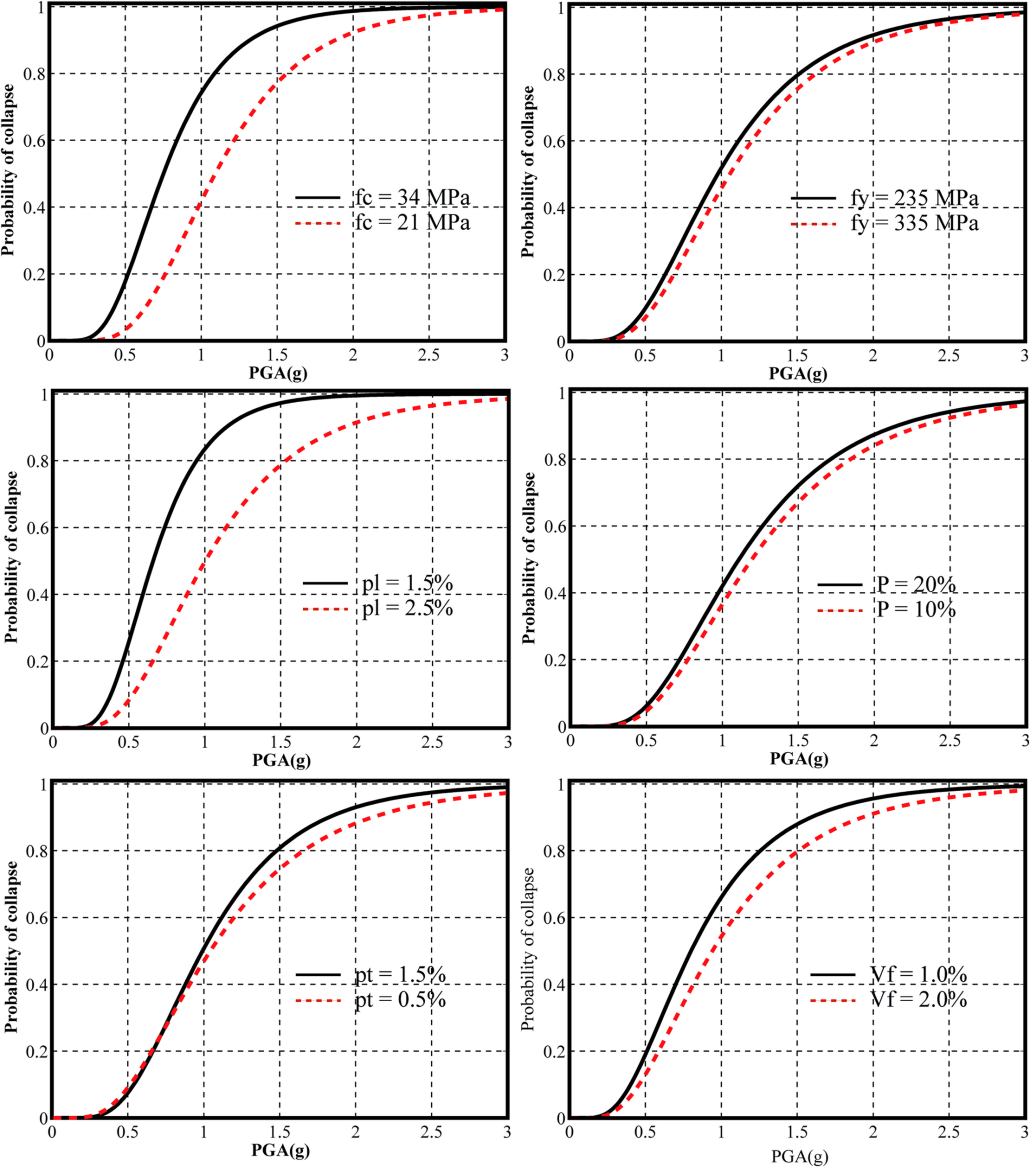
Effect of different modeling parameter on the collapse fragility curves of bridge piers.

In general, the fragility curves are usually defined as a function of IM, which describes the damage probability of structure exceeding or reaching a specific level of damage state. In the last decades, the fragility curves are usually generated by three main methods: a) the expert opinion; b) damage data observed from field information and c) numerical simulations and experiment tests. In this paper, the numerical simulation is used to generate the analytical collapse fragility curves. The IDA curves are obtained from the nonlinear time-history analyses using a suit of 20 real records scaled up until the collapse of the SFRC bridge piers. The analytical fragility curve exhibits advantages compared to other methods. When using the IDA method, the collapse fragility curves can be calculated using Eq (5) as a lognormal cumulative distribution function (CDF):

$$P(Collapse|im=x)=\Phi (\frac{\ln(x/c)}{\beta })$$ (8)

in which *P* represents the collapse probability, x is a specific conditional value of IM. Φ(.) is the CDF of standard normal distribution, *c* is the collapse median intensity of the fragility curves, and β is the standard deviation. The parameter *c* and β can be estimated by a simplified fitting method proposed by Baker [27]. Fig 9 shows the collapse fragility curves generated from the IDA results shown in Fig 7. In Fig 9, the fragility curves with black solid line give higher collapse probability, while the fragility curves with dash red line illustrate a lower collapse fragility.

### 6.1 Effect of different parameter on the collapse fragility curves

Fig 9(A) illustrates that the compressive strength of concrete has a significant effect on the collapse fragility of SFRC bridge piers based on the PGA and corresponding maximum drift ratio of top pier. The solid black fragility curve and the dash red fragility curve represent the probability of collapse with concrete strength of 34 MPa and 21 MPa respectively. It should be noted that 34 MPa pier is more fragile compared to 21 MPa pier. It can be attributed to the fact that 34 MPa pier has higher stiffness than that of 20 MPa pier, which can attract more seismic inertia forces from earthquakes and cause more seismic damages to the pier. Since concrete with lower strength (21 Mpa) has lower elasticity modulus, larger deformation of 21 MPa pier in seismic loading can activate the confinement of steel fibers and thus may be more effective in resisting seismic inertia forces when comparing to the 34 MPa pier at same level of drift ratio. It can also be seen from Fig 9(A) that the 21 MPa pier has a PGA value of 1.1 g at 50% collapse probability while the 34 MPa pier has a PGA value of only 0.72 g to experience similar damage level.

It can be observed from Fig 9(B) that the effect of steel yield strength on the collapse fragility of SFRC bridge piers is relatively small compared to the effect of concrete compressive strength. The solid black and the dash red fragility curve show the collapse probability with the steel yield strength of 335 MPa and 235 MPa respectively. From Fig 9(B), it can be seen that the small difference between collapse fragility curves of 335 MPa and 235 MPa can be due to the reason that both steel bars with 335 MPa and 235 MPa have the same elasticity modulus, which attract similar seismic forces. The 335 MPa steel has much larger strain and yield stress comparing to the 235 MPa steel, which can experience lower seismic damage level at the same seismic intensity. From Fig 9(B), it can be seen that the 335 MPa steel pier has a PGA value of 1.06 g at 50% collapse probability, whereas the 235 MPa steel pier has a PGA value of 0.97 g.

Fig 9(C) shows the longitudinal reinforcement ratio has a significant effect on the collapse fragility of SFRC bridge piers. The solid black and the dash red fragility curves illustrate the collapse probability of longitudinal reinforcement ratios of 1.5% and 2.5% respectively. Larger ratio of longitudinal reinforcement increases the stiffness and strength of the bridge pier, which can cause lesser deformation at similar seismic hazard level comparing to lower steel ratio. From Fig 9(C), it can be seen that the bridge pier with 2.5% longitudinal steel ratio has a PGA value of 1.09 g at 50% collapse probability, whereas the bridge pier with 1.5% longitudinal steel ratio has a PGA value of 0.66 g at same level of probability.

It can be observed from Fig 9(D) that the axial load ratios has a similar effect on the collapse fragility of SFRC bridge piers comparing to the effect of steel yield strength. The solid black and the dash red fragility curves illustrate the collapse probability of axial load ratios of 20% and 10% respectively. Larger ratio of axial load ratio increases the stiffness and the corresponding period of the bridge pier, which can have more seismic inertia forces and cause more seismic damage comparing to lower axial ratio. From Fig 9(D), it can be seen that the bridge pier with 10% axial load ratio has a PGA value of 1.19 g at 50% collapse probability, while the bridge pier with 20% axial load ratio has a PGA value of 1.11 g.

Fig 9(E) shows the transverse hoops ratio has minor effect on the collapse fragility of SFRC bridge piers. The solid black and the dash red fragility curves show the collapse probability of transverse hoops ratio of 1.5% and 0.5%respectively. From Fig 9(E), it can be seen that the bridge pier with 1.5% transverse hoops ratio has a PGA value of 0.99 g at 50% collapse probability, whereas the bridge pier with 0.5% transverse hoops ratio has a PGA value of 1.04 g at same level of probability.

It can be observed from Fig 9(F) that the steel fiber content has a significant effect on the collapse fragility of SFRC bridge piers. The solid black and the dash red fragility curves illustrate the collapse probability of steel fiber content of 1% and 2% respectively. Larger ratio of steel fiber content increases the stiffness and strength of the bridge pier, which can cause lesser deformation at similar seismic hazard level comparing to lower steel fiber content. From Fig 9(F), it can be seen that the bridge pier with 1% steel fiber content has a PGA value of 0.81 g at 50% collapse probability, whereas the bridge pier with 2% steel fiber content has a PGA value of 0.95 g at same level of probability.

### 6.2 Median collapse fragility

**Fig 10:**
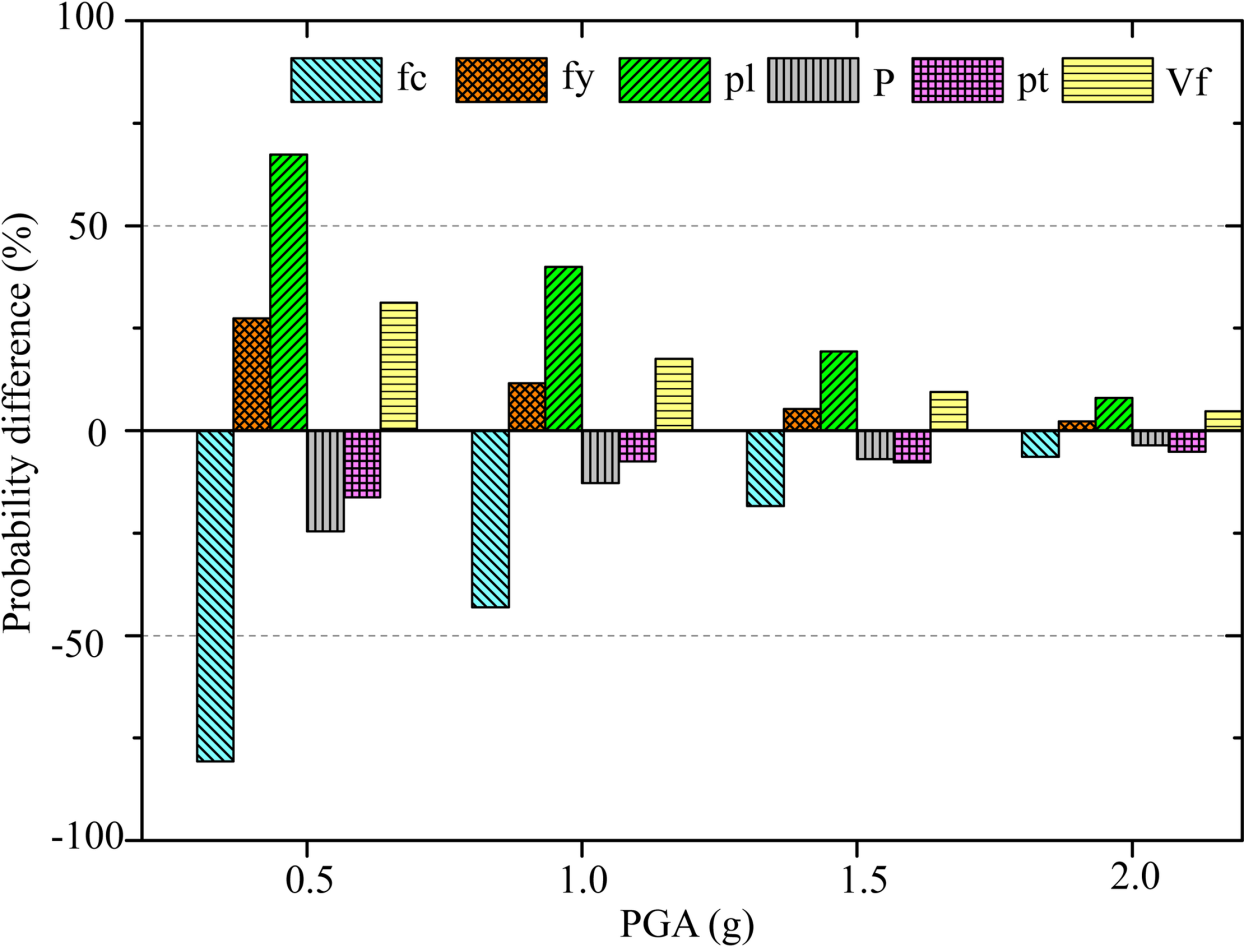
Collapse probability difference between different parameters.

The probability difference for collapse fragilities of different parameters are shown in a bar chart in Fig 10 for four seismic hazard levels (PGA = 0.5 g, 1.0 g, 1.5 g, and 2.0 g respectively). It can be seen from Fig 10 that the differences in collapse probability gradually decrease when the PGA increases. In other words, the uncertainties from ground motions are the major source of uncertainties when the seismic hazard is at a high level. It can also be seen from Fig 10 that the sensitivity of each parameter can be found at each PGA value. In Fig 10, the concrete compressive strength and longitudinal reinforcement ratio are the significant parameters.

**Fig 11:**
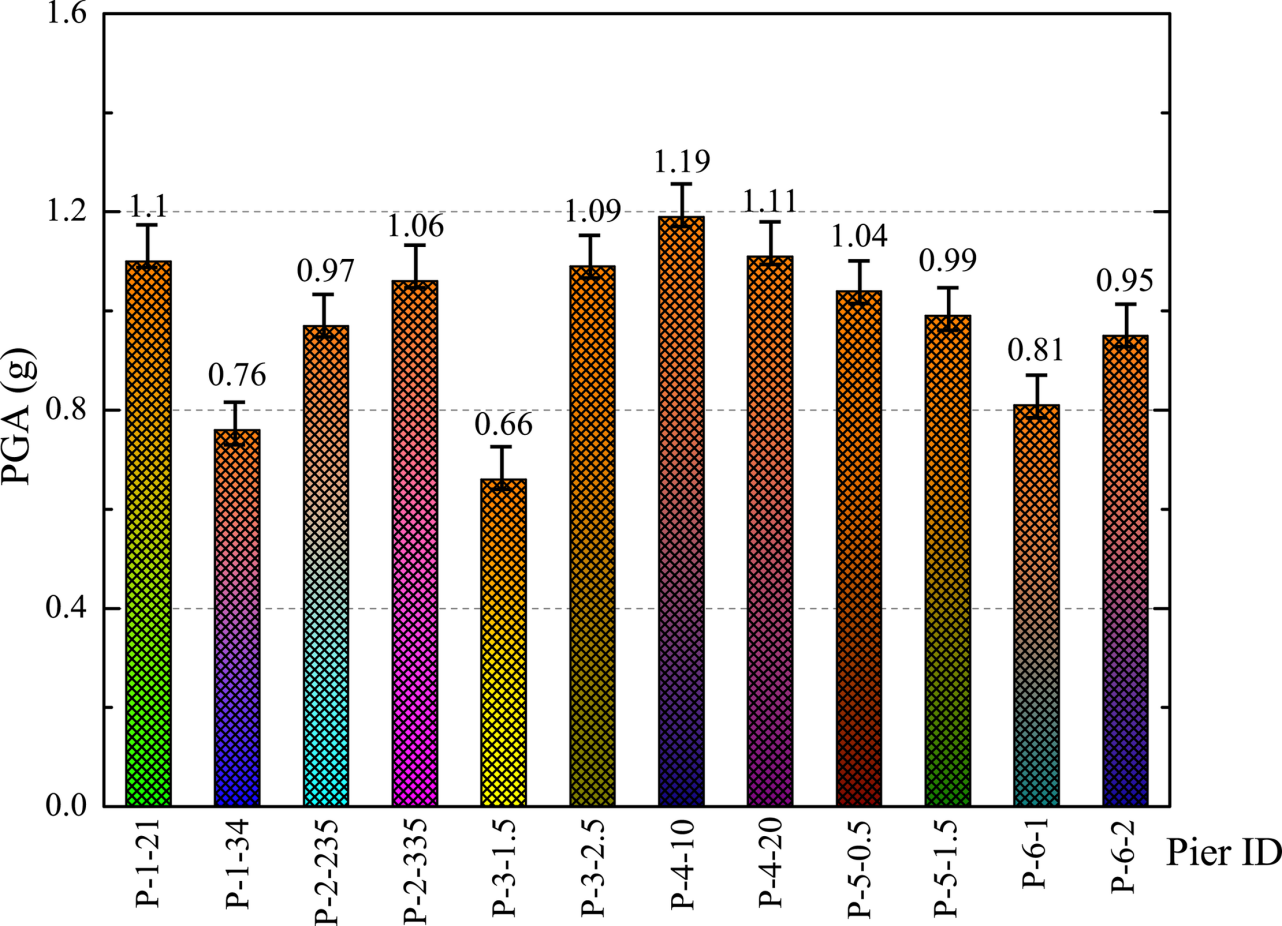
Median fragilities for bridge piers with different parameters.

The median values of PGA for collapse fragility curves of different parameters of SFRC bridge piers are given in Fig 11. The median values of PGA are calculated from probability in the collapse fragility curves reaching 50%. It can be expected that lower median value of one pier is more fragile compared to higher median values of other piers. For example, SFRC bridge piers with longitudinal reinforcement ratio of 1.5% have the lowest value of median, 0.66 g, which indicate the worst seismic performance of SFRC bridge piers. It should be noted that the SFRC bridge pier with 21 MPa concrete shows higher median value of 1.1 g. It indicates that the steel fibers are more effective in 21 MPa concrete compared to 34 MPa concrete (PGA = 0.76 g). Thus, it is concluded that the seismic performance of bridge piers can be improved with steel fibers when the concrete compressive strength is not very high.

## Conclusions

The seismic collapse assessment of the SFRC piers are evaluated through fragility curves, which is a popular tool to estimate the damage probability of bridges at different levels of seismic hazards. Since the piers are the most important and vulnerable component in the bridge system, the present paper uses the nonlinear static pushover analyses (NSPA) and nonlinear incremental dynamic analyses (IDA) to generate the collapse fragility curves of SFRC bridge piers located in China with different modeling parameters. Based on the results, the following conclusions can be drawn:

1) Higher values of different parameters can increase the capacity of SFRC bridge piers. 21 MPa concrete decreases the base shear at the yield, crushing, buckling and fracture limit states by 12.8%, 3.7%, 3.8% and 3.9% comparing to 34 MPa concrete. Higher yield steel strength (335 MPa) has much higher base shear by 12.4%, 22.6%, 21.6% and 21.5% comparing to 235 MPa steel yield force. The yielding, crushing, buckling and fracture base shear capacity can be improved by 32.0%, 31.2%, 31.4% and 31.3% when the pier uses 2.5% longitudinal steel ratio. The base shear capacity at 10% axial load ratio decrease by 12.1%, 4.9%, 5.0% and 5.1%. The maximum effect of transverse hoops ratio on the base shear capacity of SFRC piers is almost 3%. The steel fiber content has 1.5%, 11.5%, 19.4% and 19.6% larger yielding, crushing, buckling and fracture base shear capacities comparing to the 1% steel fiber content.

2) Different parameters have different influence on the collapse fragility curves of SFRC piers conditional on the PGA. The 21 MPa pier has lower collapse probability at a PGA value of 1.1 g comparing to the PGA value of 0.72 g of 34 MPa pier. The 335 MPa steel pier has lower collapse probability at a PGA value of 1.06 g comparing to the PGA value of 0.97 g of the 235 MPa steel pier. The bridge pier with 2.5% longitudinal steel ratio has lower collapse probability at a PGA value of 1.09 g comparing to the PGA value of 0.66 g of the bridge pier with 1.5% longitudinal steel ratio. The bridge pier with 10% axial load ratio has higher collapse probability at a PGA value of 1.19 g comparing to the PGA value of 1.11 g of the bridge pier with 20% axial load ratio. The bridge pier with 1.5% transverse hoops ratio has higher collapse probability at a PGA value of 0.99 g comparing to the PGA value of 1.04 g of the bridge pier with 0.5% transverse hoops ratio. The bridge pier with 1% steel fiber content has lower collapse probability at a PGA value of 0.81 g comparing to the PGA value of 0.95 g of the bridge pier with 2% steel fiber content.

### Recommendations for practice and future research

From the results in this paper, it can be concluded that steel fibers are effective for improving the flexural capacity of piers when the piers have low reinforcement strength and low longitudinal reinforcement ratio. The concrete strength, axial load ratio and transverse hoop ratio have minor effect. Thus, if someone wants to design SFRC piers for bridges with relatively low steel strength and longitudinal steel ratio, then the steel fiber is the good option for improving the flexural capacity of bridge piers. Moreover, the collapse probability of SFRC bridge piers is significantly influenced by the concrete compressive strength, steel fiber contents and longitudinal steel ratio. The yield strength of steel, axial load ratio and transverse hoop ratio have relatively small effect on the collapse fragility of SFRC piers. So it is better for bridge engineers to use the steel fiber in the bridge pier with relatively high concrete strength and longitudinal steel ratio in order to reduce the seismic risk of bridges located in high seismic zones.

There are many directions where future research in this field can be oriented. For instance, although this study investigates the effect of six factors on the collapse performance of SFRC piers, other factors, such as strain rates, transverse reinforcement space and steel fiber types and so on, may also have significant effect on the flexural capacity and collapse ability of SFRC bridge piers. Further studies are required to include these parameters, in order to better understand the overall behavior and performance of bridge with SFRC piers. Moreover, it is better to calibrate the IDA curves of SFRC piers obtained in this paper using the shaking table tests. The large scaling in the IDA method may cause the unrealistic seismic responses, which is still a challenge for future work. In addition, current finite element models used in this paper cannot consider the effect of different distribution patterns of steel fibers in the piers. So full 3D FE models should be applied to explore this effect.

## Supporting information

S1 File The data is used in “numerical simulation” section. (TXT)

S2 File The data is used in “numerical simulation” section. (TXT)

S3 File The data is used in “numerical simulation” section. (TXT)

S4 File The data is used in “numerical simulation” section. (TXT)

S5 File The data is used in “nonlinear static pushover analysis” section. (TXT)

S6 File The data is used in “incremental dynamic analysis” section. (TXT)

S7 File The data is used in “collapse fragility curves” section. (TXT)

S8 File The data is used in “collapse fragility curves” section. (TXT)

S1 Table Collapse PGA and drift of piers based on IDA results. (DOCX)
