# Supplementary material for: Seismic collapse assessment of bridge piers constructed with steel fibers reinforced concrete
Source: PLoS One. 2018 Jul 10;13(7):e0200072. doi: 10.1371/journal.pone.0200072 (PMC6039000; doi:10.1371/journal.pone.0200072)
Supplement: S1 Table — (DOCX) [file pone.0200072.s009.docx]

Table A Collapse PGA and drift of piers based on IDA results

| Ground motions | Strength of cover concrete | | | | Yield strength of steel | | | | Longitudinal reinforcements ratio | | | |
| --- | --- | --- | --- | --- | --- | --- | --- | --- | --- | --- | --- | --- |
|  | 34 MPa | | 21 MPa | | 235 MPa | | 335 MPa | | 1.5% | | 2.5% | |
|  | PGA(g) | Drift(%) | PGA(g) | Drift(%) | PGA(g) | Drift(%) | PGA(g) | Drift(%) | PGA(g) | Drift(%) | PGA(g) | Drift(%) |
| 'Imperial Valley-06' | 1.60 | 8.40 | 1.75 | 9.74 | 1.89 | 7.13 | 1.39 | 8.83 | 1.39 | 8.32 | 2.03 | 11.87 |
| 'Hector Mine' | 1.24 | 8.11 | 1.60 | 16.09 | 1.67 | 16.78 | 1.53 | 19.42 | 0.81 | 9.21 | 1.67 | 16.67 |
| 'Duzce, Turkey' | 0.59 | 7.81 | 0.74 | 10.03 | 1.24 | 18.00 | 1.17 | 19.20 | 0.67 | 10.83 | 0.95 | 16.81 |
| 'Chi-Chi, Taiwan' | 0.74 | 8.27 | 1.17 | 18.19 | 1.09 | 12.44 | 1.24 | 19.56 | 0.45 | 5.49 | 1.39 | 18.09 |
| 'Chi-Chi, Taiwan-06' | 0.67 | 7.71 | 1.11 | 12.07 | 0.95 | 7.87 | 0.67 | 8.04 | 0.59 | 7.47 | 0.31 | 3.74 |
| 'Coyote Lake' | 0.73 | 8.20 | 0.88 | 16.74 | 0.74 | 6.40 | 1.03 | 19.13 | 0.59 | 10.29 | 0.74 | 7.15 |
| 'Loma Prieta' | 0.88 | 7.63 | 1.53 | 15.30 | 1.03 | 7.56 | 1.60 | 12.85 | 0.74 | 6.82 | 1.31 | 13.67 |
| 'Chi-Chi, Taiwan' | 0.52 | 7.25 | 1.10 | 13.53 | 0.59 | 7.11 | 0.67 | 7.65 | 0.45 | 8.72 | 1.24 | 15.07 |
| 'Kocaeli, Turkey' | 1.10 | 7.52 | 1.46 | 14.80 | 1.03 | 6.16 | 1.53 | 16.01 | 0.81 | 5.61 | 1.24 | 7.78 |
| 'Chi-Chi, Taiwan' | 0.52 | 12.65 | 0.52 | 12.98 | 0.52 | 14.54 | 0.38 | 4.91 | 0.52 | 18.29 | 0.59 | 14.95 |
| 'Loma Prieta' | 1.75 | 5.61 | 2.25 | 10.82 | 2.61 | 16.92 | 2.47 | 14.83 | 1.67 | 7.36 | 2.25 | 10.11 |
| 'Victoria, Mexico' | 1.46 | 13.20 | 1.62 | 17.98 | 1.67 | 17.92 | 1.46 | 16.73 | 1.17 | 13.84 | 1.60 | 17.92 |
| 'Northridge-01' | 0.45 | 6.03 | 1.08 | 13.03 | 0.95 | 11.60 | 1.39 | 17.13 | 0.38 | 5.99 | 1.10 | 12.40 |
| 'Loma Prieta' | 0.59 | 7.69 | 0.67 | 15.47 | 0.74 | 13.05 | 0.74 | 18.45 | 0.52 | 7.85 | 0.67 | 17.26 |
| 'Chi-Chi, Taiwan-03' | 0.38 | 2.08 | 0.74 | 15.12 | 0.31 | 3.34 | 0.81 | 12.34 | 0.31 | 3.54 | 0.45 | 3.69 |
| 'Loma Prieta' | 0.45 | 11.44 | 0.45 | 15.52 | 0.45 | 11.92 | 0.32 | 9.48 | 0.52 | 6.76 | 0.59 | 15.81 |
| 'Northridge-01' | 0.59 | 4.94 | 1.01 | 12.96 | 1.10 | 17.83 | 1.03 | 12.67 | 0.88 | 12.28 | 1.17 | 19.95 |
| 'Northridge-01' | 0.67 | 6.79 | 1.09 | 18.03 | 1.31 | 18.55 | 1.24 | 19.94 | 0.52 | 11.86 | 1.03 | 17.83 |
| 'Northridge-01' | 0.66 | 3.46 | 1.03 | 15.26 | 1.39 | 15.64 | 1.17 | 13.69 | 0.59 | 3.39 | 1.02 | 13.67 |
| 'Chi-Chi, Taiwan' | 0.95 | 6.12 | 1.82 | 9.19 | 0.74 | 3.62 | 1.67 | 9.14 | 0.88 | 6.19 | 1.05 | 6.27 |
| Ground motions | Axial load ratio | | | | Transverse hoops ratio | | | | Steel fiber content | | | |
|  | 20% | | 10% | | 0.5% | | 1.5% | | 1% | | 2% | |
|  | PGA(g) | Drift(%) | PGA(g) | Drift(%) | PGA(g) | Drift(%) | PGA(g) | Drift(%) | PGA(g) | Drift(%) | PGA(g) | Drift(%) |
| 'Imperial Valley-06' | 2.18 | 6.06 | 2.36 | 6.38 | 2.03 | 10.01 | 1.89 | 8.61 | 1.39 | 5.49 | 1.62 | 5.58 |
| 'Hector Mine' | 1.53 | 15.55 | 1.66 | 17.10 | 1.89 | 15.95 | 1.24 | 7.73 | 1.60 | 11.81 | 1.95 | 14.24 |
| 'Duzce, Turkey' | 1.10 | 15.02 | 1.42 | 17.75 | 1.10 | 18.03 | 1.06 | 18.57 | 0.81 | 10.22 | 0.95 | 9.91 |
| 'Chi-Chi, Taiwan' | 1.24 | 14.85 | 1.42 | 17.50 | 1.08 | 14.11 | 1.10 | 12.98 | 1.10 | 14.39 | 1.28 | 14.79 |
| 'Chi-Chi, Taiwan-06' | 1.18 | 13.33 | 1.03 | 7.07 | 0.95 | 7.89 | 0.95 | 7.48 | 0.52 | 6.48 | 0.61 | 4.43 |
| 'Coyote Lake' | 1.03 | 15.16 | 1.19 | 17.64 | 0.74 | 3.29 | 0.74 | 11.55 | 0.67 | 5.37 | 0.78 | 4.19 |
| 'Loma Prieta' | 2.11 | 12.51 | 1.74 | 11.56 | 0.95 | 4.70 | 1.01 | 7.56 | 1.09 | 8.00 | 1.37 | 8.40 |
| 'Chi-Chi, Taiwan' | 0.88 | 9.76 | 0.72 | 7.00 | 0.45 | 3.85 | 0.59 | 7.11 | 0.59 | 7.27 | 0.69 | 7.32 |
| 'Kocaeli, Turkey' | 1.53 | 13.38 | 1.58 | 14.24 | 1.53 | 6.32 | 1.03 | 6.16 | 0.74 | 4.38 | 1.03 | 5.02 |
| 'Chi-Chi, Taiwan' | 0.45 | 8.80 | 0.41 | 4.41 | 0.52 | 4.86 | 0.52 | 13.50 | 0.52 | 5.58 | 0.61 | 5.37 |
| 'Loma Prieta' | 2.39 | 7.56 | 2.59 | 13.10 | 3.40 | 12.52 | 2.39 | 10.13 | 2.32 | 9.41 | 2.71 | 9.14 |
| 'Victoria, Mexico' | 1.46 | 13.41 | 1.58 | 15.05 | 1.67 | 16.74 | 1.67 | 17.92 | 1.53 | 14.15 | 1.79 | 14.80 |
| 'Northridge-01' | 1.24 | 14.57 | 1.35 | 16.10 | 0.45 | 6.36 | 0.95 | 11.60 | 0.45 | 5.71 | 0.53 | 5.71 |
| 'Loma Prieta' | 0.67 | 12.14 | 0.80 | 12.98 | 0.95 | 18.97 | 0.74 | 12.77 | 0.74 | 15.95 | 0.86 | 16.22 |
| 'Chi-Chi, Taiwan-03' | 0.45 | 5.47 | 0.88 | 10.98 | 1.03 | 15.59 | 1.46 | 18.19 | 0.31 | 3.21 | 0.36 | 4.23 |
| 'Loma Prieta' | 0.38 | 9.40 | 0.33 | 9.57 | 0.59 | 10.13 | 0.45 | 9.23 | 0.45 | 4.50 | 0.53 | 4.54 |
| 'Northridge-01' | 1.03 | 15.75 | 1.11 | 16.47 | 1.31 | 13.61 | 1.10 | 17.96 | 0.88 | 8.69 | 1.03 | 8.65 |
| 'Northridge-01' | 1.17 | 14.91 | 1.35 | 17.90 | 1.17 | 13.60 | 1.31 | 19.64 | 1.10 | 9.00 | 1.28 | 9.05 |
| 'Northridge-01' | 1.15 | 12.60 | 1.27 | 13.95 | 0.52 | 3.36 | 1.39 | 20.08 | 0.38 | 3.28 | 0.36 | 4.58 |
| 'Chi-Chi, Taiwan' | 1.67 | 7.95 | 1.81 | 7.59 | 1.75 | 8.57 | 0.38 | 3.00 | 1.17 | 7.05 | 1.45 | 7.86 |
